# Supplementary material for: Vibrio parahaemolyticus T6SS2 effector repertoires
Source: Gut Microbes. 2023 Feb 19;15(1):2178795. doi: 10.1080/19490976.2023.2178795 (PMC9980498; doi:10.1080/19490976.2023.2178795)
Supplement: Supplemental Material [file KGMI_A_2178795_SM4653.zip › Supplementary Information_bioRxiv.pdf]

Supplementary Information

Vibrio parahaemolyticus T6SS2 effector repertoires

Supplementary Figures S1-S3

Supplementary Datasets S1-S3

Supplementary Tables S1-S2

Supplementary References

Supplementary Figures

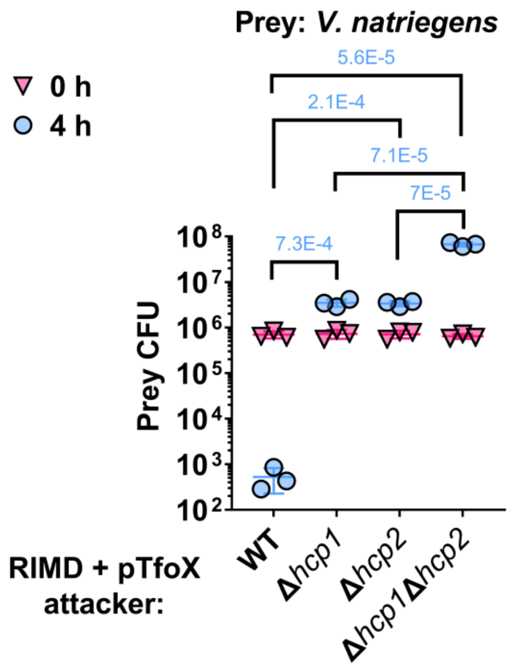

Supplementary Fig. S1. T6SS1 and T6SS2 play a role in interbacterial competition in *V. parahaemolyticus* strain RIMD 2210633. Viability counts (CFU) of the *V. natriegens* prey strain before (0 h) and after (4 h) co-incubation with the indicated *V. parahaemolyticus* RIMD 2210633 attacker strains carrying a plasmid for the arabinose-inducible expression of TfoX (pTfoX) on LB agar plates supplemented with 0.1% (wt/vol) L-arabinose to induce expression from plasmids. The statistical significance between samples at the 4 h time point was calculated using an unpaired, two-tailed Student's *t*-test. Data are shown as the mean ± SD; *n* = 3.

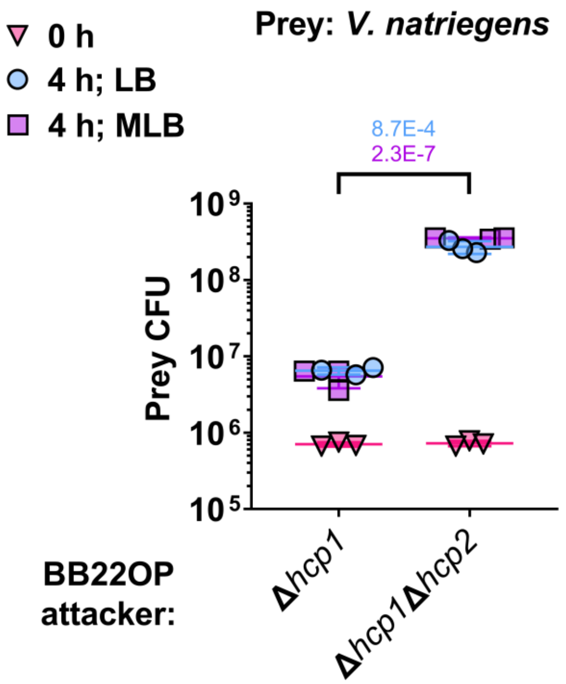

Supplementary Fig. S2. The activity of *V. parahaemolyticus* BB22OP T6SS2 is comparable in LB and MLB media. Viability counts (CFU) of *V. natriegens* prey strain before (0 h) and after (4 h) co-incubation with the indicated *V. parahaemolyticus* BB22OP attacker strains on LB or MLB agar plates. The statistical significance between samples at the 4 h time point was calculated using an unpaired, two-tailed Student's *t*-test. Data are shown as the mean ± SD; $n = 3$.

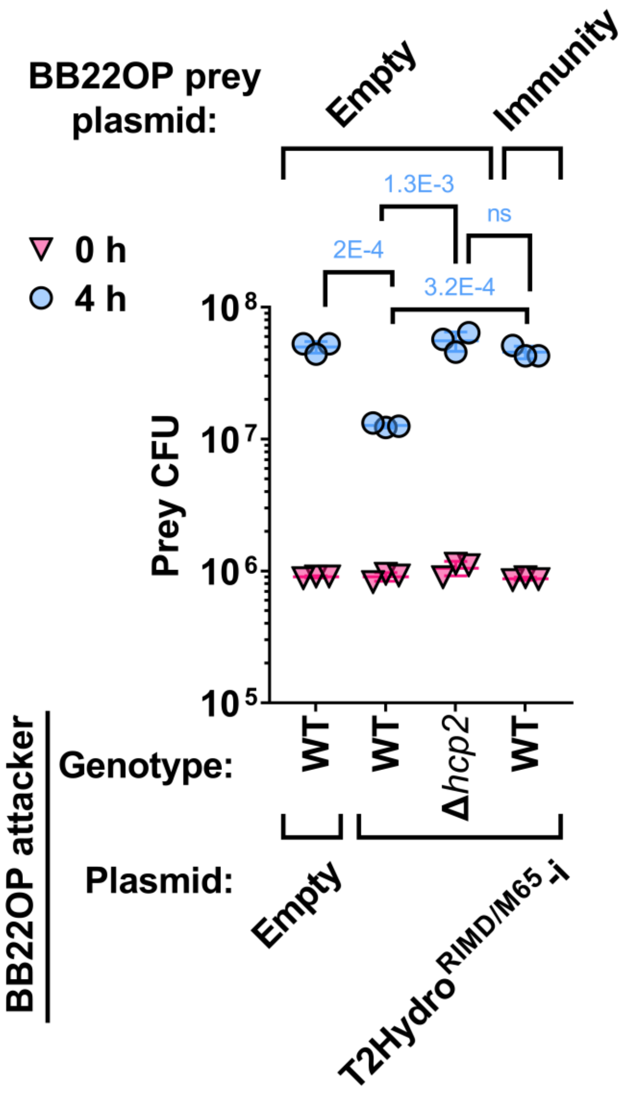

Supplementary Fig. S3. T2Hydro$^{RIMD/M65}$ is a functional T6SS2 effector. Viability counts (CFU) of *V. parahaemolyticus* BB22OP prey strains containing either an empty plasmid (Empty) or a plasmid for the arabinose-inducible expression of the T2Hydro^{RIMD} cognate protein (Immunity) before (0 h) and after (4 h) co-incubation with the indicated *V. parahaemolyticus* BB22OP attacker strains carrying an empty plasmid or a plasmid for the arabinose-inducible expression of T2Hydro$^{RIMD/M65}$ and its downstream-encoded immunity protein (T2Hydro$^{RIMD/M65}$-i) on LB agar plates supplemented with 0.1% (wt/vol) L-arabinose to induce expression from plasmids. The statistical significance between samples at the 4 h time point was calculated using an unpaired, two-tailed Student's *t*-test; ns, no significant difference ($P > 0.05$). Data are shown as the mean ± SD; $n = 3$.

Supplementary Datasets

Supplementary Dataset S1. Mass spectrometry results for the *V. parahaemolyticus* BB22OP samples.

Supplementary Dataset S2. Mass spectrometry results for the *V. parahaemolyticus* RIMD 2210633 samples.

Supplementary Dataset S3. The distribution of T6SS2 effectors in complete *V. parahaemolyticus* genomes.

Supplementary Tables

Supplementary Table S1. A list of bacterial strains used in this work.

Strain name	Genotype	Comments	Source
Vibrio parahaemolyticus RIMD 2210633	Wild type	Used for generating deletion strains, Hcp2 secretion assays, and as an attacker in competition assays	Obtained from Kim Orth
Vibrio parahaemolyticus BB22OP	Wild type	Used for generating deletion strains, Hcp2 secretion assays, proteomic analysis and as an attacker in competition assays	Obtained from Kim Orth
Vibrio natriegens ATCC 14048	Wild type	Used as prey in competition assays	ATCC collection
Vibrio parahaemolyticus RIMD 2210633 Δ*hcp1*	Δ*vp1393*	RIMD 2210633 derivative containing an in-frame deletion of *vp1393;* used in proteomic analysis and as an attacker in competition assays	(1)
Vibrio parahaemolyticus RIMD 2210633 Δ*hcp2*	Δ*vpa1027*	RIMD 2210633 derivative containing an in-frame deletion of *vpa1027*; used in secretion assays and as an attacker in competition assays	This study
Vibrio parahaemolyticus RIMD 2210633 Δ*hcp1*Δ*hcp2*	Δ*vp1393*Δ*vpa1027*	RIMD 2210633 derivative containing an in-frame deletion of *vp1393* and *vpa1027*; used in proteomic analysis and as an attacker in competition assays	This study
Vibrio parahaemolyticus RIMD 2210633 Δ*T2Rhs-Nuc*^{RIMD}	Δ*vp1517*	RIMD 2210633 derivative containing an in-frame deletion of *vp1517*; used in Hcp2 secretion assays and as an attacker in competition assays	This study

Vibrio parahaemolyticus RIMD 2210633 Δ*T2Rhs-Nuc^{RIMD}-i*	Δ*vp1517/8*	RIMD 2210633 derivative containing an in-frame deletion of *vp1517* and *vp1518*; used as prey in competition assays	This study
Vibrio parahaemolyticus RIMD 2210633 Δ*T2Hydro^{RIMD}*	Δ*vpa0347*	RIMD 2210633 derivative containing an in-frame deletion of *vpa0347*; used in Hcp2 secretion assays and as an attacker in competition assays	This study
Vibrio parahaemolyticus RIMD 2210633 Δ*T2Hydro^{RIMD}-i*	Δ*vpa0347/8*	RIMD 2210633 derivative containing an in-frame deletion of *vpa0347* and *vpa0348*; used as prey in competition assays	This study
Vibrio parahaemolyticus RIMD 2210633 Δ*T2LipB^{RIMD}*	Δ*vp0626*	RIMD 2210633 derivative containing an in-frame deletion of *vp0626*; used in Hcp2 secretion assays	This study
Vibrio parahaemolyticus RIMD 2210633 Δ*T2LipB^{RIMD}-i*	Δ*vp0626/5*	RIMD 2210633 derivative containing an in-frame deletion of *vp0626* and *vp0625*; used as prey in competition assays.	This study
Vibrio parahaemolyticus BB22OP Δ*hcp1*	Δ*vpbb_rs06665*	BB22OP derivative containing an in-frame deletion of *vpbb_rs06665;* used as an attacker in competition assays	(2)
Vibrio parahaemolyticus BB22OP Δ*hcp2*	Δ*vpbb_rs19920*	BB22OP derivative containing an in-frame deletion of *vpbb_rs19920;* used in Hcp2 secretion assays, proteomic analysis, and as an attacker in competition assays	(2)
Vibrio parahaemolyticus BB22OP Δ*hcp1*Δ*hcp2*	Δ*vpbb_rs06665*Δ*vpbb_rs19920*	BB22OP derivative containing an in-frame deletion of Δ*vpbb_rs06665* and Δ*vpbb_rs19920*; used in Hcp2 secretion assays	(2)

		and as an attacker in competition assays	
Vibrio parahaemolyticus BB22OP Δ*T2Rhs-Nuc*^{BB22}	Δ*vpbb_rs07235*	BB22OP derivative containing an in-frame deletion of *vpbb_rs07235*; used in Hcp2 secretion assays and as an attacker in competition assays	This study
Vibrio parahaemolyticus BB22OP Δ*T2Rhs-Nuc*^{BB22}*-i*	Δ*vpbb_rs07235/40*	BB22OP derivative containing an in-frame deletion of *vpbb_rs07235* and *vpbb_rs07240*; used as prey in competition assays	This study
Vibrio parahaemolyticus BB22OP Δ*T2Tme*^{BB22}	Δ*vpbb_rs07950*	BB22OP derivative containing an in-frame deletion of *vpbb_rs07950*; used in Hcp2 secretion assays and as an attacker in competition assays	This study
Vibrio parahaemolyticus BB22OP Δ*T2Tme*^{BB22}*-i*	Δ*vpbb_rs07950/25090*	BB22OP derivative containing an in-frame deletion of *vpbb_rs07950* and *vpbb_rs25090*; used as prey in competition assays	This study
Vibrio parahaemolyticus BB22OP Δ*T2Unkwn*^{BB22}	Δ*vpbb_rs18835*	BB22OP derivative containing an in-frame deletion of *vpbb_rs18835*; used in Hcp2 secretion assays and as an attacker in competition assays	This study
Vibrio parahaemolyticus BB22OP Δ*T2Unkwn*^{BB22}*-i*	Δ*vpbb_rs18835/40*	BB22OP derivative containing an in-frame deletion of *vpbb_rs18835* and *vpbb_rs18840*; used as prey in competition assays	This study
Vibrio parahaemolyticus BB22OP Δ*T2LipA*^{BB22}	Δ*vpbb_rs22630*	BB22OP derivative containing an in-frame deletion of *vpbb_rs22630*; used in Hcp2 secretion assays	This study

		and as an attacker in competition assays	
Vibrio parahaemolyticus BB22OP Δ*T2LipA^{BB22}-i*	Δ*vpbb_rs22630/25*	BB22OP derivative containing an in-frame deletion of *vpbb_rs22630* and *vpbb_rs22625*; used as prey in competition assays	This study
Vibrio parahaemolyticus BB22OP Δ*T2LipB^{BB22}*	Δ*vpbb_rs03020*	BB22OP derivative containing an in-frame deletion of *vpbb_rs22630*; used in Hcp2 secretion assays	This study
Vibrio parahaemolyticus BB22OP Δ*T2LipB^{BB22}-i*	Δ*vpbb_rs03020/15*	BB22OP derivative containing an in-frame deletion of *vpbb_rs03020* and *vpbb_rs03015*; used as prey in competition assays	This study
Escherichia coli DH5α (*λ-pir*)	K-12 derivative laboratory strain containing λ-pir	Used for plasmid maintenance and cloning	Obtained from Eric V. Stabb

Supplementary Table S2. A list of plasmids used in this work.

**Plasmid name**	**Description**	**Purpose**	**Source**
pBAD^{K}/Myc-His	pBR322 ori-containing plasmid harboring a Kan^{R} cassette, *araC*, and an MCS following a P*bad* promoter	Used for arabinose-inducible expression	(3)
pBAD18	Bacterial plasmid with Gentamycin resistance	Used to provide selectable resistance to *V. parahaemolyticus* prey strains in competition assays	Addgene
pBAD33	Bacterial plasmid with chloramphenicol resistance	Used to provide selectable resistance to *V. natriegens* prey strains in competition assays	Addgene
pBAD33.1^{F}	pBAD33.1 with a FLAG tag inserted at the 3' end of the MCS	Used for arabinose-inducible expression of proteins	(2)

pTfoX	pBAD^{K}/Myc-His plasmid containing the CDS of TfoX (VP1241) from *V. parahaemolyticus* RIMD 2210633 in frame with a C-terminal Myc-His tag	Used for the arabinose-inducible expression of TfoX	(4)
pImmunity$^{T2Rhs\text{-}NucRIMD}$	pBAD33.1^{F} containing the CDS of the T2Rhs-Nuc^{RIMD} immunity protein (VP1518) from *V. parahaemolyticus* RIMD 2210633 in-frame with a C-terminal Flag tag	Used for the arabinose-inducible expression of the T2Rhs-Nuc^{RIMD} immunity protein	This study
pImmunity^{T2HydroRIMD}	pBAD33.1^{F} plasmid containing the CDS of the T2Hydro^{RIMD} immunity protein (VPA0348) from *V. parahaemolyticus* RIMD 2210633 in-frame with a C-terminal Flag tag	Used for the arabinose-inducible expression of the T2Hydro^{RIMD} immunity protein	This study
pT2Hydro$^{RIMD/M65}$-i	pBAD33.1^{F} plasmid containing the CDS of T2Rhs-Nuc$^{RIMD/M65}$ and its immunity protein (VPA0347 starting with methaionine 65 and VPA0348) from *V. parahaemolyticus* RIMD 2210633 in-frame with a C-terminal Flag tag	Used for the arabinose inducible expression of the pT2Hydro$^{RIMD/M65}$-i effector/immunity pair in BB22OP surrogate attacker strains in competition assays	This study
pImmunity$^{T2Rhs\text{-}NucBB22}$	pBAD33.1^{F} plasmid containing the CDS of the T2Rhs-Nuc^{BB22} immunity protein (VPBB_RS07240) from *V. parahaemolyticus* BB22OP in-frame with a C-terminal Flag tag	Used for the arabinose-inducible expression of the T2Rhs-Nuc^{BB22} immunity protein	This study
pImmunity^{T2TmeBB22}	pBAD33.1^{F} plasmid containing the CDS of the T2Tme^{BB22} immunity protein (VPBB_RS25090) from *V. parahaemolyticus*	Used for the arabinose-inducible expression of the T2Tme^{BB22} immunity protein	This study

	BB22OP in-frame with a C-terminal Flag tag		
pImmunity^{T2UnkwnBB22}	pBAD33.1^{F} plasmid containing the CDS of the T2Unkwn^{BB22} immunity protein (VPBB_RS18840) from *V. parahaemolyticus* BB22OP in-frame with a C-terminal Flag tag	Used for the arabinose-inducible expression of the T2Unkwn^{BB22} immunity protein	This study
pImmunity^{T2LipABB22}	pBAD33.1^{F} plasmid containing the CDS of the T2LipA^{BB22} immunity protein (VPBB_RS22625) from *V. parahaemolyticus* BB22OP in-frame with a C-terminal Flag tag	Used for the arabinose-inducible expression of the T2LipA^{BB22} immunity protein	This study
pDM4	a Cm^{R} and ori$_{R6K}$-containing suicide vector	Used to generate deletions in *Vibrio*	(5)
pDM4:*hcp1*^{RIMD}	pDM4 containing 1 kb downstream and 1 kb upstream of *vp1393* in its MCS	Used to delete *hcp1* in *V. parahaemolyticus* RIMD 2210633	(3)
pDM4:*hcp2*^{RIMD}	pDM4 containing 1 kb upstream and 1 kb downstream of *vpa1027* in its MCS	Used to delete *hcp2* in *V. parahaemolyticus* RIMD 2210633	This study
pDM4:*hcp1*^{BB22}	pDM4 containing 1 kb upstream and 1 kb downstream of *vpbb_rs06665* in its MCS	Used to delete *hcp1* in *V. parahaemolyticus* BB22OP	(2)
pDM4:*hcp2*^{BB22}	pDM4 containing 1 kb upstream and 1 kb downstream of *vpbb_rs19920* in its MCS	Used to delete *hcp2* in *V. parahaemolyticus* BB22OP	(2)
pDM4:*T2Rhs-Nuc*^{RIMD}	pDM4 containing 1 kb upstream and 1 kb downstream of *vp1517* in its MCS	Used to delete *T2Rhs-Nuc*^{RIMD} in *V. parahaemolyticus* RIMD 2210633	This study
pDM4:*T2Rhs-Nuc*^{RIMD}*-i*	pDM4 containing 1 kb upstream of *vp1517* and 1 kb downstream of *vp1518* in its MCS	Used to delete *T2Rhs-Nuc*^{RIMD} and its downstream immunity gene in *V. parahaemolyticus* RIMD 2210633	This study

pDM4:*T2Hydro*^{RIMD}	pDM4 containing 1 kb upstream and 1 kb downstream of *vpa0347* in its MCS	Used to delete *T2Hydro*^{RIMD} in *V. parahaemolyticus* RIMD 2210633	This study
pDM4:*T2Hydro*^{RIMD}*-i*	pDM4 containing 1 kb upstream of *vpa0347* and 1 kb downstream of *vpa0348* in its MCS	Used to delete *T2Hydro*^{RIMD} and its downstream immunity gene in *V. parahaemolyticus* RIMD 2210633	This study
pDM4:*T2LipB*^{RIMD}	pDM4 containing 1 kb upstream and 1 kb downstream of *vp0626* in its MCS	Used to delete *T2LipB*^{RIMD} in *V. parahaemolyticus* RIMD 2210633	This study
pDM4:*T2LipB*^{RIMD}*-i*	pDM4 containing 1 kb upstream of *vp0625* and 1 kb downstream of *vp0626* in its MCS	Used to delete *T2LipB*^{RIMD} and its upstream gene in *V. parahaemolyticus* RIMD 2210633	This study
pDM4:*T2Rhs-Nuc*^{BB22}	pDM4 containing 1 kb upstream and 1 kb downstream of *vpbb_rs07235* in its MCS	Used to delete *T2Rhs-Nuc*^{BB22} in *V. parahaemolyticus* BB22OP	This study
pDM4:*T2Rhs-Nuc*$^{BB22-}$*i*	pDM4 containing 1 kb upstream and 1 kb downstream of *vpbb_rs07235/40* in its MCS	Used to delete *T2Rhs-Nuc*^{BB22} and its downstream immunity gene in *V. parahaemolyticus* BB22OP	This study
pDM4:*T2Tme*^{BB22}	pDM4 containing 1 kb upstream and 1 kb downstream of *vpbb_rs07950* in its MCS	Used to delete *T2Tme*^{BB22} in *V. parahaemolyticus* BB22OP	This study
pDM4:*T2Tme*^{BB22}*-i*	pDM4 containing 1 kb upstream of *vpbb_rs07950* and 1 kb downstream of *vpbb_rs25090* in its MCS	Used to delete *T2Tme*^{BB22} and its downstream immunity gene in *V. parahaemolyticus* BB22OP	This study
pDM4:*T2Unkwn*^{BB22}	pDM4 containing 1 kb upstream and 1 kb downstream of *vpbb_rs18835* in its MCS	Used to delete *T2Unkwn*^{BB22} in *V. parahaemolyticus* BB22OP	This study
pDM4:*T2Unkwn*^{BB22}*-i*	pDM4 containing 1 kb upstream of *vpbb_rs18835* and 1 kb downstream of	Used to delete *T2Unkwn*^{BB22} and its downstream immunity gene in *V.*	This study

	vpbb_rs18840 in its MCS	*parahaemolyticus* BB22OP	
pDM4:*T2LipA*^{BB22}	pDM4 containing 1 kb upstream and 1 kb downstream of *vpbb_rs22630* in its MCS	Used to delete *T2LipA*^{BB22} in *V. parahaemolyticus* BB22OP	This study
pDM4:*T2LipA*^{BB22}*-i*	pDM4 containing 1 kb upstream of *vpbb_rs22625* and 1 kb downstream of *vpbb_rs22630* in its MCS	Used to delete *T2LipA*^{BB22} and its upstream immunity gene in *V. parahaemolyticus* BB22OP	This study
pDM4:*T2LipB*^{BB22}	pDM4 containing 1 kb upstream and 1 kb downstream of *vpbb_rs03020* in its MCS	Used to delete *T2LipB*^{BB22} in *V. parahaemolyticus* BB22OP	This study
pDM4:*T2LipB*^{BB22}*-i*	pDM4 containing 1 kb upstream of *vpbb_rs03015* and 1 kb downstream of *vpbb_rs03020* in its MCS	Used to delete *T2LipB*^{BB22} and its upstream gene in *V. parahaemolyticus* BB22OP	This study

Supplementary References

1. Dar Y, Jana B, Bosis E, Salomon D. 2022. A binary effector module secreted by a type VI secretion system. EMBO Rep 23:e53981.

2. Fridman CM, Keppel K, Gerlic M, Bosis E, Salomon D. 2020. A comparative genomics methodology reveals a widespread family of membrane-disrupting T6SS effectors. Nat Commun 11:1085.

3. Salomon D, Gonzalez H, Updegraff BL, Orth K. 2013. Vibrio parahaemolyticus Type VI secretion system 1 Is activated in marine conditions to target bacteria, and is differentially regulated from system 2. PLoS One 8:e61086.

4. Ben-Yaakov R, Salomon D. 2019. The regulatory network of Vibrio parahaemolyticus type VI secretion system 1. Environ Microbiol 21:2248–2260.

5. O’Toole R, Milton DL, Wolf-Watz H. 1996. Chemotactic motility is required for invasion of the host by the fish pathogen Vibrio anguillarum. Mol Microbiol 19:625–637.
